# Morphological control of heterostructured nanowires synthesized by sol-flame method

**DOI:** 10.1186/1556-276X-8-347

**Published:** 2013-08-08

**Authors:** Runlai Luo, In Sun Cho, Yunzhe Feng, Lili Cai, Pratap M Rao, Xiaolin Zheng

**Affiliations:** 1 Department of Materials Science and Engineering, Stanford University, Stanford, CA 94305, USA; 2 Department of Mechanical Engineering, Stanford University, Stanford, CA 94305, USA

**Keywords:** Heterostructured nanowires, Metal oxide nanowires, Sol-flame, Flame synthesis, CuO nanowires, Co_3_O_4_ nanoparticles

## Abstract

Heterostructured nanowires, such as core/shell nanowires and nanoparticle-decorated nanowires, are versatile building blocks for a wide range of applications because they integrate dissimilar materials at the nanometer scale to achieve unique functionalities. The sol-flame method is a new, rapid, low-cost, versatile, and scalable method for the synthesis of heterostructured nanowires, in which arrays of nanowires are decorated with other materials in the form of shells or chains of nanoparticles. In a typical sol-flame synthesis, nanowires are dip-coated with a solution containing precursors of the materials to be decorated, then dried in air, and subsequently heated in the post-flame region of a flame at high temperature (over 900°C) for only a few seconds. Here, we report the effects of the precursor solution on the final morphology of the heterostructured nanowire using Co_3_O_4_ decorated CuO nanowires as a model system. When a volatile cobalt salt precursor is used with sufficient residual solvent, both solvent and cobalt precursor evaporate during the flame annealing step, leading to the formation of Co_3_O_4_ nanoparticle chains by a gas-solid transition. The length of the nanoparticle chains is mainly controlled by the temperature of combustion of the solvent. On the other hand, when a non-volatile cobalt salt precursor is used, only the solvent evaporates and the cobalt salt is converted to nanoparticles by a liquid–solid transition, forming a conformal Co_3_O_4_ shell. This study facilitates the use of the sol-flame method for synthesizing heterostructured nanowires with controlled morphologies to satisfy the needs of diverse applications.

## Background

**Figure 1:**
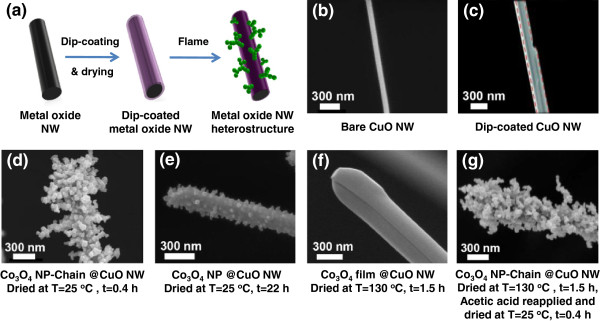
**Effects of solvent on the morphology of Co**_**3**_**O**_**4 **_**on CuO NWs.** Schematic drawing of the sol-flame method **(a)**, for which bare CuO NWs **(b)** are dip-coated with a cobalt precursor containing cobalt salt and solvent and air dried **(c)**, followed by a rapid flame annealing process to form Co_3_O_4_-decorated CuO NW heterostructure. SEM image of Co_3_O_4_-decorated CuO NWs prepared by the sol-flame method with different air-drying conditions: 25°C for 0.4 h **(d)**, 25°C for 22 h **(e)**, 130°C for 1.5 h **(f)**, and first dried at 130°C for 1.5 h, then reapplied acetic acid and dried at 25°C for 0.4 h **(g)**. Extensive drying by increasing duration or temperature inhibits the formation of the Co_3_O_4_ NP-chain morphology.

Heterostructured nanowires (NWs), such as radially modulated core/shell NWs, axially modulated NWs, nanoparticle (NP)-decorated NWs, and branched NWs, are of great interest for diverse applications because they integrate dissimilar materials at the nanometer length scale on individual NWs to achieve unique and unprecedented functionalities [1-7]. Heterostructured NWs have already demonstrated their potential in applications such as photoelectrochemistry [8,9], catalysis [10], sensors [11,12], and batteries [13,14]. For instance, Ge/Si core/shell NW field-effect transistors achieve much higher performance than planar Si metal-oxide-semiconductor field-effect transistors due to one-dimensional quantum confinement effect [15]. In addition, InP NWs, for which the depletion regions are filled with InAsP quantum dots, showed an increase of carrier gain of four orders of magnitude per absorbed photon compared to a conventional diode structure as single-photo detectors [16]. Moreover, branched TiO_2_ NWs for photoelectrochemical water-splitting exhibited an incident photon-to-current conversion efficiency that is two times higher than that of bare TiO_2_ NWs resulting from increased surface area and improved charge separation and transport within the branches [17]. Controlled and scalable synthesis of heterostructured NWs is a critical prerequisite for their broad applications. Heterostructured NWs are currently synthesized by methods such as the sol–gel method [18], hydrothermal method [13], physical/chemical vapor deposition [19], and self-assembly [20]. Our group has recently developed a new sol-flame method (Figure 1a), which combines solution chemistry and rapid flame annealing to decorate NWs with other materials in the form of shells or chains of NPs to form heterostructured NWs [21-23]. Compared to other existing methods, the sol-flame method has the unique and important advantages of rapid material growth rate, low cost, versatility and scalability. Previously, we investigated the effect of flame annealing temperature on the final morphology of the heterostructured NWs and found that high temperature flame annealing leads to NP-chain formation and low temperature favors shell formation on the NWs. In this paper, we investigate the effects of solution chemical compositions on the morphology of the heterostructured NWs synthesized by the sol-flame method. We use copper (II) oxide (CuO) NWs decorated by cobalt (II, III) oxide (Co_3_O_4_) as a model system because both CuO and Co_3_O_4_ are important materials for catalysis and electrochemical applications and hence control of their composites and nanostructures during the synthesis is critical to improve their properties [24-28]. We study the dependence of the final morphology of the decorated Co_3_O_4_ on the chemical compositions of the solvent and the cobalt salt used in the cobalt precursor solution.

## Methods

### Synthesis of CuO NWs

CuO NWs are first synthesized by a thermal annealing method [29-32], where copper wires (wire diameter 0.0045 in.; McMaster, Atlanta, GA, USA) with a length of 1 cm are annealed at 550°C for 12 h in air in a tube furnace (Lindberg/Blue M, Waltham, MA, USA) to grow CuO NWs perpendicularly to the copper wire surface.

### Preparation of cobalt precursor solutions

The cobalt precursor solutions with a typical concentration of 0.04 M are prepared by mixing cobalt acetate tetrahydrate (Co(CH_3_COO)_2_·4H_2_O, 99%, Sigma-Aldrich Chemicals, St. Louis, MO, USA) or cobalt nitrate hexahydrate (Co(NO_3_)_2_·6H_2_O, 99%, Sigma-Aldrich Chemicals) with acetic acid (CH_3_COOH, 99.7%, EMD Chemicals, Merck KGaA, Darmstadt, Germany) or propionic acid (C_2_H_6_COOH, 99%, Mallinckrodt Chemicals, St. Louis, MO, USA). After mixing the cobalt salt with the solvent, the cobalt precursor solutions are sonicated for 10 min to completely dissolve the cobalt salt and then aged overnight at room temperature before use.

### Sol-flame synthesis of Co_3_O_4_ decorated CuO NWs

The general procedure of the sol-flame method for the synthesis of heterostructured NWs was described previously [21-23] and is shown schematically in Figure 1a. Briefly, for our model system consisting of Co_3_O_4_-decorated CuO NWs, the as-grown CuO NWs (diameters: 70 to 200 nm and an average length: 16 μm) (Figure 1b) are dip-coated with the prepared cobalt precursor solution to form a shell of cobalt precursor on the CuO NWs, and then dried in air prior to flame annealing (Figure 1c). This dip-coating process is repeated three times to form a conformal cobalt precursor shell on top of CuO NWs. Finally, the dip-coated CuO NWs are annealed in the post-flame region of a premixed co-flow flame (McKenna Burner, Holthuis & Associates, Sebastopol, CA, USA) at a typical temperature of 990°C for 5 s, leading to the formation of Co_3_O_4_-decorated CuO NWs (Figures 1d,e,f,g). The formation reactions of Co_3_O_4_ nanoparticles from cobalt salt precursors (Co(CH_3_COO)_2_ and Co(NO_3_)_2_) are as follows in flame [33-35]:

$$\begin{array}{l} 3\mathrm{Co}{\left(NO_{3}\right)}_{2}\to Co_{3}O_{4}+6NO_{2}+O_{2}\;\left(\mathrm{endothermic}\right)\\ 3\mathrm{Co}{\left(CH_{3}\mathrm{COO}\right)}_{2}+25/2\;O_{2}\to Co_{3}O_{4}+12CO_{2}+9H_{2}O\left(\mathrm{exothermic}\right) \end{array}$$

The burner is operated with CH_4_ and H_2_ as fuels, and air as the oxidizer with a fuel to oxidizer equivalence ratio (*Φ*) of 0.84 (the flow rates of CH_4_, H_2_, and air are 2.05, 4.64, and 36.7 SLPM (standard liter per minute), respectively). The typical temperature of the post-flame region gas is 990°C that is measured by a K-type thermocouple (1/16 in. bead diameter, Omega Engineering, Inc., Stamford, CT, USA). The typical flame annealing time is 5 s.

### Material characterizations

The morphology, crystal structure, and elemental composition of the prepared heterostructured NWs are characterized by scanning electron microscopy (SEM, FEI XL30 Sirion, 5 kV, Hillsboro, OR, USA), transmission electron microscopy (TEM, Philips CM20 FEG, 200 kV, Amsterdam, The Netherlands), and TEM-energy dispersive X-ray spectroscopy (EDS), respectively.

## Results and discussion

### Effects of solvent on the morphology of Co_3_O_4_ on the CuO NWs

We first investigate the effect of residual solvent in the cobalt precursor on the final morphology of Co_3_O_4_. Typically, the cobalt precursor consists of cobalt acetate (Co(CH_3_COO)_2_·4H_2_O) dissolved in acetic acid (CH_3_COOH) solvent. We study the effect of residual acetic acid on the CuO NWs by varying the drying conditions immediately after the dip-coating step. We test three different drying conditions in air: (1) 0.4 h at 25°C, (2) 22 h at 25°C, and (3) 1.5 h at 130°C, corresponding to increasing amounts of solvent evaporation during the drying process and, therefore, decreasing amounts of residual solvent, and then we anneal all the samples in the same flame condition. First, when the dip-coated NW sample is dried at 25°C for 0.4 h (highest amount of residual solvent), a Co_3_O_4_ NP-chain morphology is formed on the CuO NWs after flame annealing (Figure 1d). Second, the longer drying duration of 22 h at 25°C leads to a smaller amount of residual solvent, and a monolayer coating of Co_3_O_4_ NPs is formed after flame annealing (Figure 1e). Third, the amount of residual solvent is minimized by drying at 130°C, which is higher than the boiling temperature of acetic acid (118°C) but is lower than the decomposition temperature of cobalt acetate (230°C) to avoid precursor decomposition [36]. In this case, no particles are observed at all, but instead, a conformal and dense layer of Co_3_O_4_ is coated onto CuO NWs (Figure 1f). In order to confirm the importance of the residual solvent, we reapply the solvent acetic acid by drop casting to the dip-coated NW that has been dried for 1.5 h at 130°C, and then air dry the NW again at 25°C for 0.4 h, and the NP-chain morphology is formed after flame annealing. These results clearly indicate that the amount of the residual solvent in the precursor coating layer (Figure 1c) before flame annealing has a strong impact on the final morphology of Co_3_O_4_ on the CuO NWs. A larger amount of residual solvent leads to the formation of the NP-chain morphology, and a smaller amount of residual solvent leads to the formation of shells, or equivalently a thin film coating.

**Figure 2:**
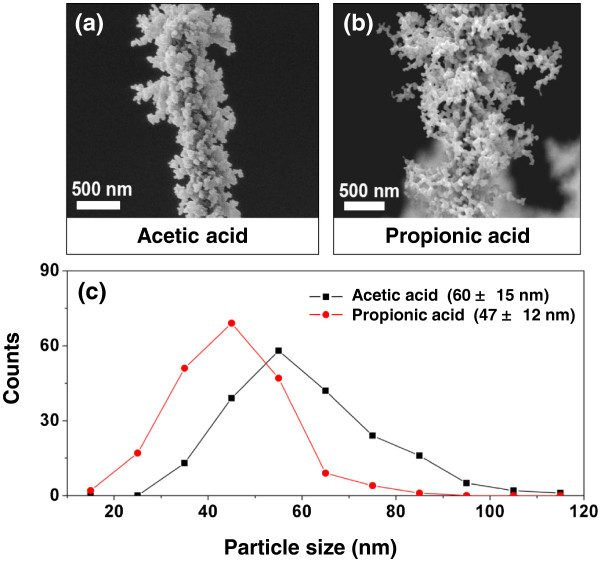
**Effects of solvent on the degree of branching and size distribution of Co**_**3**_**O**_**4 **_**NPs.** SEM images of Co_3_O_4_ NP-decorated CuO NWs synthesized using different solvents: **(a)** acetic acid and **(b)** propionic acid. **(c)** Histogram of distribution of Co_3_O_4_ NP size for these two solvents. Propionic acid has a higher temperature of combustion, resulting in a larger length of NP-chains and smaller size of the NPs compared to those resulting from the use of acetic acid.

The formation of the NP-chain morphology is due to the generation of gases by the evaporation and combustion of the coated solution on the CuO NWs during flame annealing, which induces a gas flow (i.e., Stefan flow) [23]. The above results suggest that most of the gas flow comes from the evaporation and combustion of the residual solvent rather than from the cobalt salt inside the cobalt precursor solution. To investigate the effect of solvent on the morphology of Co_3_O_4_, we select another solvent, propionic acid, to compare with acetic acid. For both solvents, the dip-coated NW samples are dried for 0.4 h at 25°C to leave a large amount of solvent on the CuO NWs before flame annealing. It is assumed that a similar amount of cobalt precursor is left on CuO NWs after drying, in each case. The use of propionic acid leads to longer NP-chains (Figure 2b) and smaller average NP size (Figure 2c) than does the use of acetic acid (Figure 2a). The length of the NP-chains increases with increasing velocity (*v*) of the gas flow which carries the cobalt acetate precursor away from the CuO NWs as it forms NPs. The induced gas velocity is determined by the mass flux ($\dot{M}$) of the evaporated solution and the density (*ρ*) of the solution vapor as v=M˙ρ. The mass flux ($\dot{M}$) of the evaporated solution depends most strongly on the temperature of solvent combustion, and the density (*ρ*) of the solution vapor is inversely proportional to the temperature of solvent combustion. Since the adiabatic combustion temperature of propionic acid (2,202 K) is higher than that of acetic acid (2,074 K), propionic acid leads to higher mass flux and lower solution vapor density, hence larger induced gas velocity. This faster induced gas flow carries cobalt acetate further away from the CuO NWs, forming longer NP-chains. The higher combustion temperature also leads to reduced gas density, which in turn reduces the gas phase concentration of cobalt acetic precursors, leading to smaller average NP size (Figure 2c). Hence, the length of the NP-chain and size of the NPs are mainly controlled by the combustion temperature of the solvent, which affects the induced gas flow velocity and the NP precursor concentration.

### Effects of cobalt salt precursor on the morphology of Co_3_O_4_ on the CuO NWs

**Figure 3:**
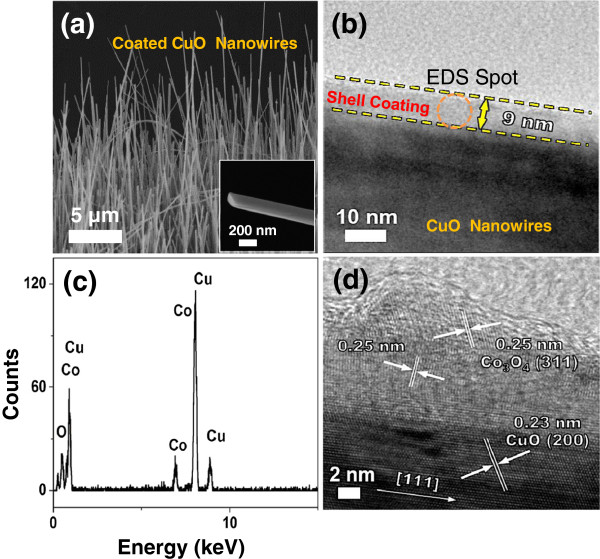
**Effects of cobalt salt precursor on the morphology of Co**_**3**_**O**_**4 **_**on CuO NWs.** A shell of Co_3_O_4_ is formed when cobalt nitrate is used as the cobalt salt precursor. **(a)** SEM image of CuO/Co_3_O_4_ core/shell NWs. The inset shows a single CuO/Co_3_O_4_ core/shell NW. **(b)** TEM image, **(c)** TEM-EDS spectrum, and **(d)** HRTEM of the CuO/Co_3_O_4_ core/shell NW edge, indicating that the shell is a 9-nm thick polycrystalline Co_3_O_4_ film.

While the morphology of Co_3_O_4_ is significantly affected by the solvent, it will also depend on the properties of the cobalt salt precursors, such as their volatility. To focus on the effect of the cobalt salt precursor, the solvent is fixed to be acetic acid with the same drying condition of 0.4 h at 25°C in air, which leaves a large amount of acetic acid in the precursor coating. We study the effect of cobalt salt precursors on the Co_3_O_4_ morphology by comparing volatile cobalt acetate Co(CH_3_COO)_2_·4H_2_O with non-volatile cobalt nitrate Co(NO_3_)_2_·6H_2_O. Volatile cobalt acetate has been used for the above control experiments and leads to the formation of the Co_3_O_4_ NP-chain morphology (Figure 1d) when there is sufficient residual solvent. When non-volatile cobalt nitrate is used as the precursor, a shell is formed on the CuO NWs instead of a NP-chain (Figure 3a), despite the presence of a large amount of residual solvent. The shell coating at the surface of the CuO NWs is about 9-nm thick (Figure 3b). The TEM-EDS analysis (Figure 3c) shows the presence of both Cu and Co peaks along with the O peak in the coated NW. Further high-resolution TEM (HRTEM) characterization (Figure 3d) reveals that the final NW consists of a single crystal CuO NW core with a [111] growth direction and a thin polycrystalline shell with an interplanar spacing of 0.25 nm, which corresponds to the spacing of (311) planes of Co_3_O_4_.

**Figure 4:**
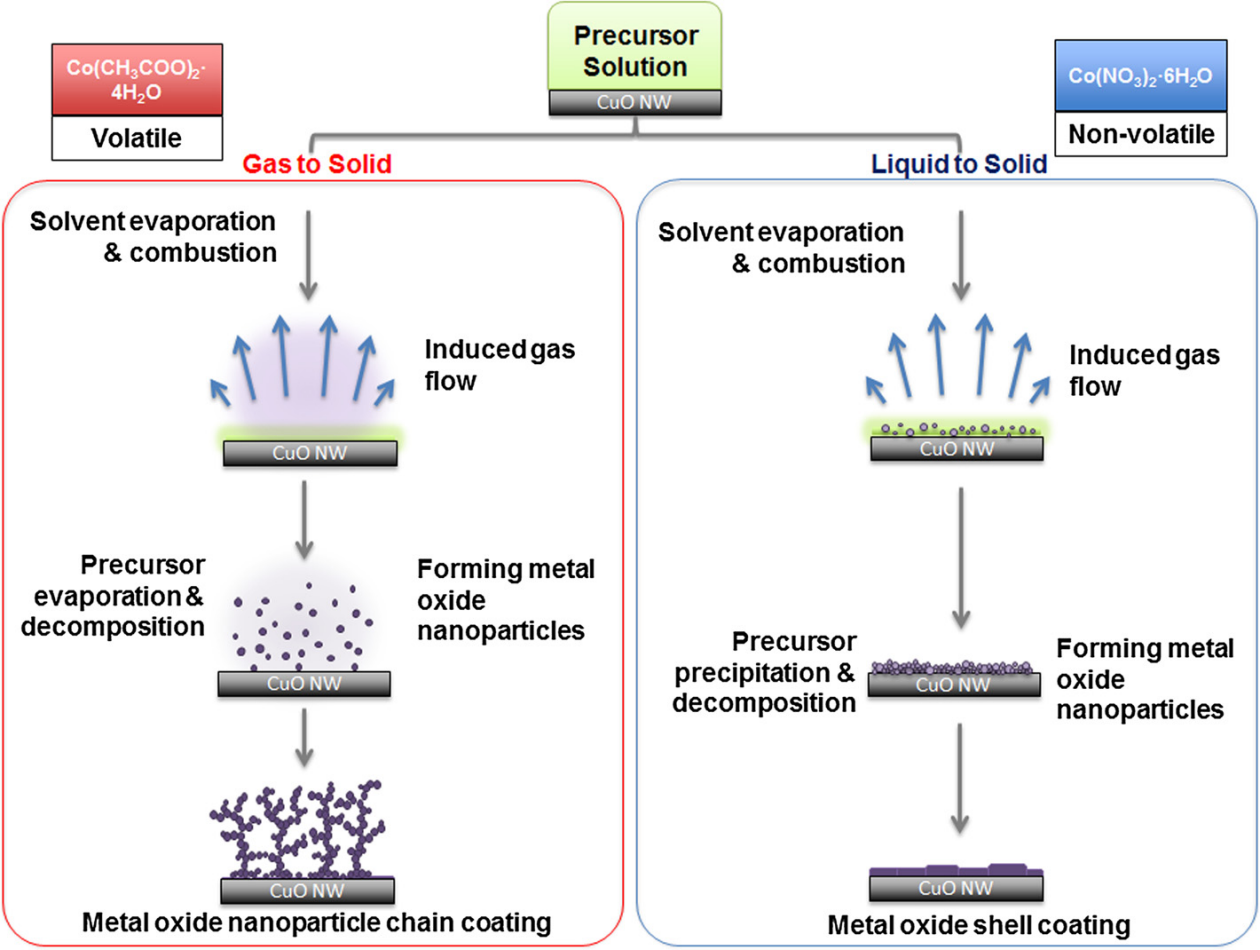
**Schematic illustration of the effects of metal salt precursor on the morphology of Co**_**3**_**O**_**4 **_**on CuO NWs.** A CuO NW is dip-coated with a cobalt precursor solution containing the solvent and cobalt salt and then annealed in the flame. (Left column) In the case of a volatile precursor (e.g., Co(CH_3_COO)_2_·4H_2_O), the precursor evaporates into vapor and nucleation of the Co_3_O_4_ occurs in the gas phase, resulting in the formation of the NP-chain morphology. (Right column) In the case of a non-volatile precursor (e.g., Co(NO_3_)_2_·6H_2_O), the precursor does not evaporate but stays in the solvent, where nucleation happens in the liquid phase, resulting in the formation of the shell morphology.

The difference in Co_3_O_4_ morphology is attributed to the difference in volatility between cobalt acetate and cobalt nitrate precursors, as described by the growth mechanism for Co_3_O_4_-decorated CuO NWs, which is schematically illustrated in Figure 4. For both cobalt salt precursors, we assume that the initial stages are the same. CuO NWs are dip-coated with the cobalt precursor solution containing both solvent and cobalt salt. After the drying step in air, approximately the same quantity of cobalt salt solution is left on the CuO NWs for both cobalt salt precursors. When the precursor-coated CuO NWs are annealed in the post-flame region of a premixed flame (990°C, 5 s), the solvent evaporates and combusts continuously and rapidly. At this stage, the volatility of the cobalt precursor affects the nucleation process. Cobalt acetate, as an organic precursor, is more volatile and evaporates together with solvent. Consequently, the nucleation of Co_3_O_4_ NPs occurs in the gas phase and is a gas-to-particle conversion process (Figure 4, left panel) [37-39]. Therefore, the length of the NP-chains is directly affected by the induced gas flow velocity. In contrast, cobalt nitrate, as an inorganic precursor, is non-volatile and has high solubility in acetic acid. Consequently, cobalt nitrate will mostly remain in the liquid phase and decompose to form NPs in a liquid-to-particle conversion process (Figure 4, right panel) [39-41], leading to the formation of a shell composed of NP aggregates.

## Conclusions

To summarize, we have investigated the fundamental aspects of morphology control of heterostructured NWs synthesized by the sol-flame method for the model system of Co_3_O_4_-decorated CuO NWs. The final morphology of Co_3_O_4_ on the CuO NWs is greatly influenced by the properties of both the solvent and the cobalt salt used in the cobalt precursor solution. First, the evaporation and combustion of the solvent induces a gas flow away from the NWs that is responsible for the formation of Co_3_O_4_ NP-chains. Solvents with higher combustion temperatures produce gas flows with larger velocity, leading to the formation of longer Co_3_O_4_ NP-chains with smaller NP size. Second, the volatility of the cobalt precursor determines the precursor decomposition and nucleation process. A volatile cobalt precursor evaporates during flame annealing and converts to NPs in a gas-solid transition, forming Co_3_O_4_ NP-chains. Non-volatile cobalt precursor mainly remains in the liquid and converts to NPs in a liquid–solid transition, favoring the formation of a Co_3_O_4_ shell. Finally, we believe that this new understanding will facilitate the use of the sol-flame method for the synthesis of heterostructured NWs with tailored morphologies to satisfy the needs of diverse applications such as catalysis, sensors, solar cells, Li-ion batteries, and photosynthesis.

## Competing interests

The authors declare that they have no competing interests.

## Authors’ contributions

RLL and XLZ designed the experiments. All authors contributed to the experiment. RLL and XLZ prepared the manuscript. RLL, XLZ, ISC, YF, LC, and PMR discussed the results and commented on the manuscript. All authors read and approved the final manuscript.
